# Development of a Concurrent Treatment Technique of Ethyl Formate and Mixtures (Nitrogen, Phosphine) to Control Citrus Mealybug (*Planococcus citri*)

**DOI:** 10.3390/insects14090720

**Published:** 2023-08-22

**Authors:** Bongsu Kim, Min-Goo Park, Gil-Hah Kim, Jeong-Oh Yang

**Affiliations:** 1 Plant Quarantine Technology Center, Animal and Plant Quarantine Agency (APQA), Gimcheon 39660, Republic of Korea; bskim79@korea.kr; 2 Department of Bioenvironmental Chemistry, Jeonbuk National University, Jeonju 54896, Republic of Korea; pmg@jbnu.ac.kr; 3 Department of Plant Medicine, College of Agriculture, Life and Environment Science, Chungbuk National University, Cheongju 28644, Republic of Korea; khkim@cbnu.ac.kr; 4 Youngnam Plant Pest Surveillance and Control Centre, Animal and Plant Quarantine Agency (APQA), Busan 48943, Republic of Korea

**Keywords:** ethyl formate, phosphine, nitrogen, *Planococcus citri*, quarantine

## Abstract

**Simple Summary:**

Mealybugs (Hemiptera: Pseucoccidae) are frequently detected pests when importing fresh commodities. Ethyl formate, a methyl bromide alternative fumigant, has been applied to imported bananas in Korea to eliminate mealybugs, but it represents a considerable cost for production and management. A liquid ethyl formate treatment technology was newly developed to reduce the cost, and the efficacy of this technology against *Planococcus citri*, a highly important species of mealybug in Korea, was evaluated in this study. In addition, the effect of enhancing the control against *P. citri* was evaluated through the simultaneous treatment of ethyl formate and phosphine. The results show that, for the treatment with ethyl formate + nitrogen, the control effect did not decrease with the addition, and fruit damage did not occur as the nitrogen concentration increased. In the case of the combined treatment with phosphine, the control effect was greatly increased even when a small amount (0.1 g/m^3^) was applied. The results indicate that the new technology can be used to control mealybugs on banana fruits, and a combined treatment of ethyl formate and phosphine can also enhance the efficacy against mealybugs.

**Abstract:**

Currently, the fumigant ethyl formate (EF) is stored as a liquified gas in metal cylinders mixed with carbon dioxide (CO_2_), but this product type is expensive to manufacture, transport, and maintain in cylinders. To address these problems, we developed a new EF fumigation technique with a nitrogen (N_2_) carrier. In this report, the susceptibility of citrus mealybugs, one of the most resistant mealybugs to fumigants, to EF was assessed; the phytotoxicity of an EF + N_2_ concurrent treatment applied to banana fruit was examined to evaluate the efficacy compared to the current EF + CO_2_ product; and the increased efficacy with a phosphine (PH_3_) addition to EF + N_2_ was also assessed. Concurrent treatment of EF and N_2_ was performed at an LC_50_ concentration of EF. N_2_ was applied in seven doses from concentrations of 79% to 95%. The phytotoxicity of EF to bananas was assessed by applying EF at 35 mg/L with N_2_ at 79%, and the color, sugar content, and weight loss of bananas were measured for 14 days after treatment. The EF with N_2_ treatment resulted in more than 50% mortality at all growth stages of the mealybug, and there was no significant difference between the untreated and treated banana fruits. EF mixed with PH_3_ showed a higher efficacy than treatment with EF alone, but only a slight increase in efficacy was observed when the PH_3_ concentration increased. These results indicate that concurrent treatment with EF and N_2_ can be used to control mealybugs on banana fruits, and combined treatment with EF and PH_3_ can also enhance the efficacy against mealybugs.

## 1. Introduction

*Dysmicoccus neobrevipes* (Beardsley), *Aspidiotus excisus* (Green), and *Dysmicoccus brevipes* (Cockerell) have been detected frequently when importing bananas to Korea. Therefore, the development of a proper treatment method using a fumigant, a gas-type insecticide, is essential for eliminating mealybugs in commodities. *Planococcus citri* Risso (Hemiptera: Pseucoccidae) is known as a highly important species of mealybug in Korea [1]. In addition, when testing fumigants against mealybugs, *P. citri* was reported to have the highest insecticide resistance and has been previously studied [2]. For these reasons, *P. citri* has been commonly used to test the efficacy of fumigants in Korea.

A fumigant is a synthetic chemical that, at the required temperature and pressure, exists in a gaseous state in sufficient concentrations to be lethal to a target pest [3]. Methyl bromide (MB), which has been used as a plant quarantine fumigant, has been designated as an ozone-layer-depleting substance under the Montreal Protocol enacted in 1989 [4]. In 2008, the IPPC (International Plant Protection Convention) adopted a recommendation for the use of an alternative to MB for plant quarantines [5]. MB not only causes the destruction of the ozone layer, but also causes damage to fresh plants such as imported and exported fruits, and related problems with disinfection have been documented [6,7]. MB causes chronic poisoning in people who perform the disinfection treatment, as well as environmental destruction and toxicity, raising the issue of human safety [8]. Due to problems such as the destruction of the ozone layer by MB, plant damage, and human safety, research on the development of alternative fumigants to MB has been actively conducted worldwide [8,9]. As replacement fumigants for MB, many studies have been conducted on ethyl formate (EF) and phosphine (PH_3_) in Korea.

Currently, EF fumigants (Vapormate, BOC Ltd., Wagga, Australia) used in Korea are in the form of a mixture of carbon dioxide (83.3%) and ethyl formate (16.7%). EF is a plant volatile substance that has insecticidal effects [10,11,12]. Many synergistic effects have been reported when the ratio of carbon dioxide is increased [13]. The EF fumigant is a fumigant that shows an insecticidal effect when pests breathe after being treated with EF in a gaseous state in an enclosed space. PH_3_ has been extensively used against insects, especially storage pests [8,14]. PH_3_ can control pests with a long-term treatment [15,16,17,18], has good penetration without affecting the quality of various grains, and is adsorbed onto plant or leaf residues. Because it does not cause contamination, PH_3_ is used worldwide for storage pest control [15,19,20].

These two fumigants have been set as the standards for the disinfection treatment of fruits upon import in Korean plant quarantine, and are used in the field according to import requirements [21]. Since both fumigants contain highly explosive substances such as active ingredients, they are manufactured in the form of a high-pressure cylinder containing a large amount of carbon dioxide (83.4% CO_2_ in EF fumigants, 98% CO_2_ in PH_3_ fumigants). That is, the currently used EF fumigant is applied by adding CO_2_ into a high-pressure gas, spraying liquid EF using a dip tube in a gas pipe, and then vaporizing it through a vaporizer, and a PH_3_ fumigator uses a similar method. Generally, these fumigants are stored in cylinders in the form of a high-pressure gas because they contain CO_2_ [22]. Such a high-pressure gas cylinder represents a considerable cost for their production, management and safety during movement of the high-pressure gas. Therefore, it is necessary to compensate for the disadvantages of the existing method and diversify the use of fumigants.

In this study, to supplement the existing cylinder method used to replace MB and diversify plant quarantine fumigants, liquid EF, which was recently developed, was vaporized in a vaporizer, and nonflammable gases, such as nitrogen (N_2_), were connected to the outside of the vaporizer and dosed. Using this technology [23], efficacy against *P. citri* was evaluated, and the phytotoxicity was evaluated on imported bananas. In addition, the effect of enhancing the control against scale insects was evaluated through the simultaneous treatment of EF fumigants and PH_3_ fumigants.

## 2. Materials and Methods

### 2.1. Insects and Bananas

Stock colonies of the citrus mealybug, *P. citri*, were successively reared on eyed potatoes at 25 ± 1 °C at a relative humidity of 60% and a photoperiod of 16:8 h (L:D) at the Plant Quarantine Technology Center in South Korea.

Six boxes of banana (13 kg per box, Dole Food Company, Dublin, Ireland) samples imported from the Philippines were stored at 13 °C until use. The average size of each banana was 500 ± 10 g.

### 2.2. Chemicals

EF (97%, Sigma–Aldrich, St. Louis, MO, USA) was of analytical grade and purchased from Aldrich Chemical Company Inc. (St. Louis, MO, USA). PH_3_ was obtained as ECO_2_Fume™ (2% PH_3_ + 98% CO_2_) from Cytec (Sydney, Australia). N_2_ was of high purity grade and purchased from SJ GasTech Inc. (Gumi, Republic of Korea).

### 2.3. Fumigants and Nitrogen Treatments

EF and PH_3_ were used to evaluate fumigant susceptibility to *P. citri*. EF was in the form of liquid EF (EF, 97%) and PH_3_ was in the form of ECO_2_Fume (2% PH_3_ + 98% CO_2_).

In the EF and N_2_ mixture treatment performed to investigate the effect of N_2_ during EF treatment, an OXYBABY gas analyzer (Wittgas, Witten, Germany) was used to measure and adjust the N_2_ concentration in a small-scale fumigation chamber (55 L), and a medium-scale (0.5 m^3^) N_2_ mixture processor (SafeFUME, Daegu, Republic of Korea) was used for liquid EF vaporization and diffusion in fumigation when evaluating banana toxicity. The N_2_ mixture processor was composed of a metal mesh net and heater for vaporizing the mixture of liquid EF and nitrogen gas, a controller for adjusting the heating temperature and heating time, and an outlet for discharging the vaporized mixed gas.

### 2.4. Fumigation

A 55 L fumigation system (UBNC, Incheon, Republic of Korea) was used to evaluate the fumigant efficacy for *P. citris*. The 55 L fumigation system consisted of a gas injection, sampling port, gas exhaust valve and pressure gauge, and a handle stopper for sealing was installed on the outside. Inside the fumigator, a miniature fan (Φ 10 cm, UBNC, Incheon, Republic of Korea) was placed for the diffusion and circulation of the fumigant.

A 0.5 m^3^ (0.5 × 1.25 × 0.8 m^3^) stainless steel fumigation chamber was used to investigate the weakening of bananas according to EF and N_2_ mixture treatment, and the 0.5 m^3^ stainless steel fumigation chamber was installed in a temperature-controllable container (13 ± 1 °C) and then carried out.

### 2.5. Inspector Preparation, Fumigator Treatment, and Insecticidal Rate Investigation

The fumigant efficacy of *P. citri* was evaluated on eggs, nymphs, and adults. Filter paper (Advantec, Tokyo, Japan) was laid in the insect breeding dish (Φ 5.5 cm × 1.5 cm, SPL, Pocheon, Republic of Korea), distilled water was applied for moisture supply, and potatoes were sliced thinly and then inoculated for each brood.

Fumigant treatment was performed at the Plant Quarantine Technology Development Center of the Agriculture, Forestry and Livestock Quarantine Headquarters. In a fumigation room, at a temperature of 13 ± 1 °C and a relative humidity of 60 ± 10%, the prepared mealybugs were placed in a 55 L desiccator (UBNC, Incheon, Republic of Korea) and then fumigated. The insecticidal rate was observed after 72 h of breeding under the same conditions as the subculture after treatment with a fumigant. The insecticidal rate was investigated using a stereomicroscope (MDG33, Leica, Wetzlar, Germany), and if there was no movement or only very weak movement when touched with a fine needle, the specimen was considered dead. All experiments were performed in triplicate for 4 h.

To measure the state change of bananas according to the fumigant treatment, a fumigant toxicity study was conducted. For fumigant resistance, bananas (Dole Food Company) were placed in a 0.5 m^3^ stainless fumigation chamber, and 35 mg/L EF and N_2_ (80, 83, 85, 90, 93, 95%) were mixed and treated. At 1, 7, and 14 days after fumigant treatment, the internal (peeled body) and external (outer skin) color, sugar content, and weight loss rate according to weight change in bananas were investigated.

### 2.6. Measurement of Fumigant Concentration

To determine the actual concentration of the fumigation treatment, monitoring was conducted for all experimental groups, and 60 mL samples were extracted per experimental group using a 60 mL syringe in a 1 L Tedlar bag (SKC, Dorset, UK). The samples were taken and monitored at 30 min and 1, 2, and 4 h after fumigation.

The concentration of EF was measured using an Agilent GC 7890A equipped with a flame ionization detector (FID) after separation on an Rtx-5 column (15 m × 250 μm × 1 μm, RESTEK, Bellefonte, PA, USA) operating in the split mode (10:1). The PH_3_ concentration was determined using an Agilent GC 7890A that was equipped with a flame photometric detector (FPD) and HP-PLOT/Q (30 m × 530 μm × 40 μm, Agilent, Santa Clara, CA, USA) operating in a split mode (10:1). The injector and oven temperature were 200 °C. The detector temperature was 250 °C. The injection volumes and flow rates of EF and PH_3_ were 60 and 20 μL and 1.5 and 5 mL/min, respectively. The concentrations of EF and PH_3_ were calculated based on peak areas against external standards.

### 2.7. Measurement of Fumigant Concentration × Time (CT)

The fumigant concentration was calculated using an equation outlined by Monro [24], based on the monitoring results performed at regular intervals.

CT = ∑(Ci + Ci + 1) (ti + 1 − ti)/2

where

C is the fumigant concentration (mg/L);
t is the time of exposure (h);
i is the order of measurement;
Ct is the concentration × time (mg h/L).

### 2.8. Statistical Analysis

Based on the results of EF, EF + N_2_ and EF + PH_3_ fumigant susceptibility targeting *P. citris*, the significance test by concentration was performed using an ANOVA with Tukey’s HSD test method using the SPSS program (IBM Corporation, Armonk, NY, USA). The LCT_50_ and LCT_99_ values were obtained using the Probit analysis [25] program.

## 3. Results

### 3.1. Evaluation of the Susceptibility of P. citri to Liquid EF Treatment

The fumigant efficacy was evaluated when liquid EF treatment was performed on the adults, nymphs, and eggs of *P. citri*. *P. citri* adults showed a 100% insecticidal effect when treated with 30.0 mg/L liquid EF for 4 h. At this time, the CT product (concentration × time) was 100.68 mg h/L (Table 1). Nymphs also showed a 100% insecticidal effect against the CT value, similar to adult insects. However, to control the eggs, a 100% insecticidal effect was shown when 70.0 mg/L liquid EF was added for 4 h, and at this time, the CT was 193.67 mg h/L (Table 1).

Statistical analysis was performed using lethal CT on the results of fumigant efficacy evaluation for all forms of *P. citri* (Table 2). As a result of comparing the lethal dose LCT_50_ for each fetus, the order of egg (43.946) > adult (39.244) > nymph (19.921) was found. Additionally, the LCT_90_ and LCT_99_ values showed a similar tendency to the LCT_50_ value, which were in the order of eggs (80.339, 131.388) > adults (72.369, 119.193) > nymphs (53.170, 118.383).

### 3.2. Evaluation of Susceptibility to P. citris according to the N_2_ Concentration during Simultaneous Treatment with Liquid EF and N_2_

The test was conducted at the LCT_50_ concentration of liquid EF (adult; 39.244, nymph; 19.921, egg; 43.946) and at six N_2_ concentrations of 80, 83, 85, 90, 93, and 95%. The insecticidal effect toward *P. citri* adults was significant and increased as the N_2_ concentration increased; the insecticidal effect reached 100% when the N_2_ concentration was 95%. There was no significant difference in the insecticidal effect between the eggs and nymphs with a change in N_2_ concentration (Table 3).

### 3.3. Evaluation of the Susceptibility of P. citris to Liquid EF and PH_3_ Treatment

The fumigant efficacy was evaluated when liquid EF and PH_3_ were combined with the adults, nymphs, and eggs of *P. citri*. *P. citri* adults showed a 100% insecticidal effect when treated with 8.0 mg/L liquid EF and 0.1 mg/L PH_3_ for 4 h (Table 4). When PH_3_ was applied at 0.1 mg/L, the LCT_50_ and LCT_99_ values of EF were 2.475 and 12.216 mg h/L, respectively (Table 5). Nymphs showed a 100% insecticidal effect when treated with 10.0 mg/L liquid ethyl formate and 0.1 mg/L phosphine for 4 h (Table 4). When phosphine was treated at 0.1 mg/L, the ethyl formate LCT50 and LCT99 values were 0.060 and 29.660 mg h/L, respectively (Table 5). However, eggs did not show a 100% insecticidal effect when treated with 50 mg/L ethyl formate based on 0.1 mg/L phosphine for 4 h. Treatment with 30.0 mg/L liquid ethyl formate and 0.5 mg/L phosphine showed 100% insecticidal effect when treated for 4 h (Table 6). When phosphine was applied at 0.5 mg/L, the LCT50 and LCT99 values of ethyl formate were 20.063 and 48.824 mg h/L, respectively (Table 5). A 100% insecticidal effect was shown when the phosphine concentration was 1.0 mg/L and the ethyl formate concentration was 30 mg/L with 4 h of treatment (Table 6). When phosphine was applied at 1.0 mg/L, the ethyl formate LCT50 and LCT99 values were 2.280 and 40.474 mg h/L, respectively (Table 5).

### 3.4. Evaluation of the Phytotoxicity of Imported Bananas when Treated with Liquid Ethyl Formate and Nitrogen

After treating imported bananas with liquid ethyl formate and nitrogen in parallel, phytotoxicity to bananas was evaluated at 1, 7, and 14 days (Table 7). In the case of external chromaticity, there was no significant difference between the treatment group and the untreated group until 7 days after treatment, but a slight change was observed in the EF treatment group compared to the untreated group after 14 days. In the case of internal chromaticity, there was no significant difference between the untreated and treated groups. At 1, 7, and 14 days after treatment, there was no significant difference in the sugar content and fruit weight loss between the treated and untreated groups.

## 4. Discussion

As an alternative to methyl bromide, Korea mainly uses two fumigants and applies them as alternatives at quarantine sites. These fumigants are an ethyl formate fumigant (EF 16.7%, CO_2_ 83.3%) and a phosphine fumigant (PH_3_ 2%, CO_2_ 98%). The combined fumigant was produced in the form of a mixture of CO_2_ to suppress the flammability of EF or the explosiveness of PH_3_. In this form, the cylinder is relatively thick to hold the high pressure of CO_2_, and the weight of the cylinder increase when the thickness increases. The weight of the basic ball cylinder reaches 50–70 kg. The technology for safely manufacturing cylinders increases the price of this fumigant, making it less economical. In addition, heavy cylinders limit portability in the field. The use of a large amount of CO_2_ creates the problem of CO_2_ emission, which negates the purpose of replacing MB as an ozone-depleting substance [26]. Vaporizing liquid EF by using N_2_ is a method of separating liquid EF and N_2_ and using it for disinfection treatment. Liquid EF is filled into a CO_2_ cylinder that is currently in use. This is a method that improves movement safety compared to the method of using a mobile device [17,18,27,28,29]. In this study, EF was injected into a vaporizer in a liquid form rather than a gas form, vaporized, and then injected into the fumigation chamber using N_2_ gas. In addition to reducing CO_2_ emissions, this method is reported to be able to process chemicals in a short time and disinfect relatively inexpensively compared to the conventional method [30].

As a result of the test, liquid EF treatment showed that it was more difficult to control *P. citri* eggs than nymphs and adults. When mixing EF and N_2_, the control effect on adults increased as the N_2_ concentration increased, but the control effect on the nymphs and eggs was not increased. Generally, pest eggs are known to be difficult to control with fumigants due to their low respiratory rate [31]. In the case of *P. citri*, it is also known that egg control is difficult [32]. Accordingly, it has been determined that all scale insects are controlled only when the concentration of eggs is completely controlled. On the other hand, the reason why the control effect of adult embryos increased with increasing N_2_ treatment concentration seems to be that adult embryos are more affected by oxygen concentration than other embryos [33,34]. In addition, Jamieson et al. [35] reported that the insecticidal effect significantly increased when EF was applied under ultra-low oxygen conditions. An increase in insecticidal effect has also been reported for other gases, and there is a report confirming that the insecticidal effect of rice pests increases as the CO_2_ concentration increases (5, 10, 20%) when mixing EF and CO_2_ [13]. However, since there is no enhanced insecticidal effect on eggs with the highest resistance, it seems difficult to reduce the dose compared to existing cylinder products during N_2_ mixing treatment. As a result of this study, it was determined that N_2_ can be used as a mixed-treatment gas because the control effect did not decrease with addition, and damage did not occur as the N_2_ concentration increased during liquid EF treatment.

In the case of PH_3_ treatment in parallel to enhance the efficacy of liquid EF, the control effect was greatly increased in all embryos, even when a small amount (0.1 g/m^3^) was applied, and the control effect was especially increased for eggs. Yang et al. [17,18] confirmed the increase in the control effect of scale insects in pineapple when treated with EF and PH_3_ in a cylinder formulation, and Cho et al. [36] also reported the enhancement of the control effect when treated in parallel against two species of mealybugs of the genus *Pseudococcus* that attack seedlings. In the results of this study, a synergistic effect can be confirmed, so it seems that liquid EF can enhance the control effect when mixed with EF + PH_3_ under the same cylinder formulation conditions. For field application of the EF and N_2_ mixture developed in this study, an evaluation of imported bananas was carried out. Other quality indicators, such as color, did not show a significant difference compared to untreated plants, so it was determined that combined liquid EF and N_2_ treatment can be used at plant quarantine disinfection treatment sites. It has been reported that liquid EF fumigant can be applied to imported bananas [29]. Additionally, Yang et al. [17,18,27,28] reported that the combined treatment of EF and N_2_ was effective for reproduction. These results suggest that it can be used as a substitute for MB in disinfection and quarantine sites.

## 5. Conclusions

The results indicate that concurrent treatment with EF and N_2_ can be used to control mealybugs in banana fruits, and combined treatment with EF and PH_3_ can also enhance efficacy against mealybugs.

## Figures and Tables

**Table 1:** Mortality of *P. citri* (adults, nymphs, and eggs) exposed to liquid EF fumigant for 4 h at 13 °C in a 55 L fumigation chamber.

Liquid EF Concentration (mg/L)	CT (mg h/L)	Adults		Nymphs		Eggs	
		*n*	Mortality ± SE ^a^ (%)	*n*	Mortality ± SE ^a^ (%)	*n*	Mortality ± SE ^a^ (%)
Control	0.00	140	0.00 ± 0.00 a ^c^	319	0.00 ± 0.00 a	78	1.33 ± 1.33 a
1.5	6.30	- ^b^	-	173	8.28 ± 1.91 a	-	-
6.0	19.38	155	8.95 ± 3.07 a	131	46.93 ± 12.52 b	-	-
10.0	22.85	-	-	-	-	134	10.40 ± 4.84 a
12.0	31.99	-	-	-	-	84	23.70 ± 2.60 b
15.0	51.09	143	63.67 ± 7.16 b	162	85.09 ± 3.91 c	-	-
30.0	100.68	154	100.00 ± 0.00 c	185	100.00 ± 0.00 c	-	-
40.0	112.65	-	-	-	-	136	97.78 ± 2.22 c
60.0	192.18	148	100.00 ± 0.00 c	217	100.00 ± 0.00 c	-	-
70.0	193.67	-	-	-	-	133	100.00 ± 0.00 c

^a^ SE is the standard error. ^b^ This concentration was not determined. ^c^ Mean values within each column followed by the same letter are not significantly different (*p* < 0.05).

**Table 2:** Lethal concentration time (LCT) of citrus *P. citri* (adult, nymph, and egg) exposed to liquid EF fumigant at 13 °C in a 55 L fumigation chamber.

Stage	*n*	LCT_50_ (h) (95% CL ^a^)	LCT_90_ (h) (95% CL)	LCT_99_ (h) (95% CL)	Slope (±SE)
Adult	740	39.244 (15.992–55.618)	72.369 (49.901–116.490)	119.193 (82.976–323.723)	4.823 ± 0.662
Nymph	1187	19.921 (13.260–26.405)	53.170 (41.801–68.909)	118.383 (88.478–181.474)	3.006 ± 0.247
Egg	565	43.946 (31.501–54.623)	80.339 (65.457–103.177)	131.388 (102.448–201.013)	4.892 ± 0.390

^a^ CL denotes the confidence limit.

**Table 3:** Mortality of *P. citri* (adults, nymphs, and eggs) exposed to liquid EF fumigant + nitrogen (N_2_) for 4 h in a 13 °C fumigation chamber.

N_2_ Ratio (%)	EF Concentration (mg/L)					
	14		7		16	
	Adults		Nymphs		Eggs	
	*n*	Mortality ± SE ^a^ (%)	*n*	Mortality ± SE ^a^ (%)	*n*	Mortality ± SE ^a^ (%)
79	89	14.64 ± 5.92 a ^b^	283	83.90 ± 2.00 a	178	84.06 ± 4.87 a
80	88	48.65 ± 9.01 b	205	86.23 ± 10.62 a	151	89.09 ± 6.62 a
83	87	50.62 ± 7.72 b	255	90.06 ± 3.43 a	107	88.83 ± 7.34 a
85	84	59.38 ± 3.77 b	360	98.82 ± 0.64 a	107	79.21 ± 12.54 a
90	90	63.33 ± 7.70 b	341	97.41 ± 1.41 a	163	87.09 ± 3.00 a
93	90	70.00 ± 6.94 bc	194	100.00 ± 0.00 a	145	95.43 ± 3.16 a
95	90	100.00 ± 0.00 c	183	86.89 ± 9.96 a	160	89.14 ± 6.53 a

^a^ SE is the standard error. ^b^ Mean values within each column followed by the same letter are not significantly different (*p* < 0.05).

**Table 4:** Comparison of the mortality of *P. citri* to EF over a range of concentrations and gas mixtures of EF + PH_3_ at 0.1 mg/L for the adults, nymphs, and eggs of the citrus *P. citri* under 4 h of exposure at 13 °C.

Liquid EF Concentration (mg/L)	Adults	Nymphs
	Mortality ± SE ^a^ (%)	Mortality ± SE ^a^ (%)
0.00	0.00 ± 0.00 a ^b^	0.00 ± 0.00 a
0.05	- ^c^	82.56 ± 6.65 b
0.10	-	89.56 ± 3.24 bc
1.00	45.56 ± 4.01 b	89.73 ± 1.71 bc
2.00	-	94.98 ± 2.00 bc
3.00	90.00 ± 8.39 c	98.17 ± 0.96 c
8.00	100.00 ± 0.00 c	-
10.00	100.00 ± 0.00 c	100.00 ± 0.00 c

^a^ SE is the standard error. ^b^ Mean values within each column followed by the same letter are not significantly different (*p* < 0.05). ^c^ This concentration was not performed.

**Table 5:** Lethal concentration time (LCT) of citrus *P. citri* (adults, nymphs, and eggs) exposed to liquid EF fumigant at 13 °C in a 55 L fumigation chamber.

Stage	PH_3_ Conc. (mg/L)	*n*	LCT_50_ (h) (95% CL ^a^)	LCT_99_ (h) (95% CL)	Slope (±SE)
Adults	0.1	442	2.475 (0.662–3.979)	12.216 (8.705–24.441)	3.356 ± 0.408
Nymphs	0.1	672	0.060 (0.000–0.305)	29.660 (12.485–384.726)	0.864 ± 0.173
Eggs	0.1	2068	17.113 (9.925–23.743)	161.332 (100.773–401.264)	2.388 ± 0.332
	0.5	1657	20.063 (17.482–22.385)	48.824 (40.344–67.509)	6.024 ± 0.625
	1.0	1127	2.280 (0.051–6.252)	40.474 (29.003–73.546)	1.863 ± 0.345

^a^ CL denotes the confidence limit.

**Table 6:** Comparison of the mortality of *P. citri* to EF over a range of concentrations and gas mixtures of EF + PH_3_ at 0.1 mg/L, EF + PH_3_ at 0.5 mg/L and EF + PH_3_ at 1.0 mg/L for eggs of the citrus *P. citri* under 4 h of exposure at 13 °C.

	Phosphine Conc. (mg/L)		
Liquid EF Concentration (mg/L)	0.1	0.5	1.0
	Mortality ± SE ^a^ (%)	Mortality ± SE ^a^ (%)	Mortality ± SE ^a^ (%)
0.0	13.87 ± 3.58 a ^b^	4.63 ± 4.63 a	8.65 ± 7.38 a
1.0	29.16 ± 8.55 a	5.60 ± 1.30 a	88.86 ± 4.33 b
3.0	35.26 ± 2.47 a	10.29 ± 4.13 a	-
5.0	- ^c^	29.86 ± 14.02 ab	93.70 ± 3.43 b
8.0	79.37 ± 7.58 b	66.18 ± 14.77 bc	-
10.0	87.87 ± 1.80 b	93.26 ± 4.48 c	97.65 ± 2.35 b
20.0	88.55 ± 2.47 b	-	-
30.0	97.88 ± 1.28 b	100.00 ± 0.00 c	100.00 ± 0.00 b
50.0	99.66 ± 0.34 b	-	100.00 ± 0.00 b

^a^ SE is the standard error. ^b^ Mean values within each column followed by the same letter are not significantly different (*p* < 0.05). ^c^ This concentration was not performed.

**Table 7:** Characteristics of bananas measured 1, 7, and 14 days after fumigation with EF + N_2_ fumigant at 13 °C in 0.5 m^3^.

Days ^a^	Treatment	External Color ^b^ (Mean ± SE ^c^)			Internal Color (Mean ± SE)			Sugar Content (Brix %)	Weight Loss Rate (%)
		L*	a*	b*	L*	a*	b*		
1 days	Control	66.23 ± 2.81	−6.39 ± 1.12	37.53 ± 1.32	65.24 ± 2.41	1.06 ± 0.79	23.74 ± 1.77	12.10 ± 1.07	0.00 ± 0.00
	EF + N_2_	67.16 ± 2.33	−7.16 ± 1.62	37.12 ± 1.38	70.42 ± 3.21	2.71 ± 3.46	26.39 ± 2.59	12.52 ± 1.88	0.00 ± 0.00
7 days	Control	66.07 ± 2.36	0.61 ± 1.27	41.92 ± 6.62	56.28 ± 2.89	0.51 ± 0.45	25.10 ± 2.01	20.80 ± 1.15	0.42 ± 0.01
	EF + N_2_	67.61 ± 3.25	1.61 ± 1.02	41.95 ± 5.96	61.30 ± 5.21	−0.05 ± 3.93	20.61 ± 3.79	20.62 ± 1.35	0.35 ± 0.42
14 days	Control	60.97 ± 6.03	4.06 ± 1.43	38.50 ± 6.09	55.31 ± 7.27	0.42 ± 1.47	15.06 ± 4.03	18.16 ± 2.90	0.77 ± 0.01
	EF + N_2_	54.77 ± 1.88	4.52 ± 1.65	28.90 ± 2.43	56.02 ± 5.26	0.12 ± 1.60	14.31 ± 2.34	21.26 ± 1.13	0.70 ± 0.39

^a^ Days after treatment. ^b^ Parameters for the color were based on CIE 1976 (L*, a*, b*) color space. L*: lightness, a*: green/magenta, b*: blue/yellow. ^c^ SE is the standard error.

## Data Availability

Data are contained within the article.
